# Reflections: 25th anniversary of the Institute of Biological Engineering (IBE)

**DOI:** 10.1186/s13036-020-00241-6

**Published:** 2020-07-09

**Authors:** Brahm P. Verma

**Affiliations:** College of Engineering; College of Agricultural and Environmental Sciences, University of Georgia, Athens, GA 30602-4435 USA

As the founding president of the Institute of Biological Engineering (IBE) and an IBE Fellow, let me take this opportunity to convey my most sincere congratulations and appreciation to ALL of the Institute of Biological Engineering’s current and past members, Councilors, Presidents and meeting participants, and authors of articles published in IBE’s official journal, the *Journal of Biological Engineering*, for your contributions to the advancement of the discipline of biological engineering. Here I share with you the genesis of IBE because it remains so unique and relevant, a few highlights of the past 25 years, and what is the invented future. We have come a long way since and have long ways to go.

## The genesis

On May 20, 1995, a group of seven young engineering faculty members and two student visionaries from US universities met in a two-day brainstorm session facilitated by me to reflect on the promise of integrating biology with engineering.

Ingenious solutions for practical problems - engineering - predates science as we know it today. Trial and error taught us that for solutions to work they must conform to what governs natural systems. The discovery in 1897 of the organizational unit of matter, the atom, and subsequent advances that govern the physical world increasingly provided scientific basis replacing trial and error to test the viability of proposed solutions.

Beginning in the 1930’s, the convergence of physics with engineering translated scientific explanation to manipulation of matter formalized the discipline of mechanical engineering whose inventions touch all aspects of our lives. Disciplines of chemical engineering, and electrical engineering have seen similar evolution, and they too have transformed the way we live today.

From the mid-1950’s after the discovery of DNA structure, we understand how heredity and biological information is embedded in the organizational unit of organisms, the gene. Subsequent discoveries raised the possibility of converging biology with engineering to translate scientific explanation into design of living machines and establish a new discipline of ***Biological Engineering***. For this reason, this group came together 25 years ago to deliberate on new possibilities.

The group deduced that, like the discipline of mechanical engineering whose inspiring science is physics, there is an evolving discipline of biological engineering. Although not yet perceived widely, this new discipline would be inspired by the science of biology; and it would contribute to advances in engineering sciences and design not yet envisioned.

To capture this opportunity, motions were placed substantially stating, "We form a learned society for advancing biology-inspired engineering and the 10 attending members should become members of an interim governing body, called The Council; elect Brahm Verma the Chair of the Interim Council; and name the Society: the Institute of Biological Engineering.

The Constitution and Bylaws drafted then wisely defined the purpose and the vision of the IBE stating:

*The INSTITUTE shall encourage,****in the broadest and most liberal manner****, interest and inquiry in biological engineering, fostering international cooperation among engineers, scientists, technologists, and allied professionals in order to apply engineering principles to systems of biological origin to improve the quality of the human condition*.

In subsequent years IBE engaged a wide range of expertise to develop a definition of biological engineering:

*Biological engineering is the biology-based engineering discipline that integrates life sciences with engineering in the advancement and application of fundamental concepts of biological systems from molecular to ecosystem levels.*

And IBE adopted the byline: *IBE – A Society for Advancing Biology-Inspired Engineering*.

## The first 25 years

At the start of IBE in mid-1990’s, the number of skeptics questioning validity of biological engineering as a new engineering discipline far outnumbered the believers. Resistance and lack of resources to build a professional society from scratch was simply daunting. In the midst of this time I wrote an article entitled, “*An Emerging New Order: Biological Engineering a Discipline and a Profession*” in which I quoted Machiavelli: “*There is nothing more difficult to take in hand, more perilous to conduct, or more uncertain in its process, than to take the lead in the introduction of a new order of things.*”

Machiavelli was prophetic. IBE has experienced what he stated. However, in the end IBE has come through to be a society of value whose vision remains unmatched. Many societies have added and integrated biology to their purposes, and several new societies have emerged. But IBE remains the only society that embraces biological engineering *in the broadest and most liberal manner*, thus remains a potential home for many in other related societies.

IBE has held at least one meeting annually to bring together the best of minds to share the most advanced research. High school, undergraduate, graduate and postdoctoral students present their exciting work and make life-long connections with peers. Young faculty have professionally achieved remarkable successes advancing to become distinguished chair professors, department heads, deans and a university president. IBE has provided exceptional opportunities particularly to its young members to take on leadership responsibilities and make personal connections with the connected.

A most visible and impactful achievement is the establishment of the *Journal of Biological Engineering*. The publication mimics IBE vision by publishing articles that liberally cover the breadth of the discipline.

IBE remain a most relevant society after 25 years and continues to seek a bright future to serve its vision.

**Next 25 Years – “*The best approach to predict the future is to invent it.”***

In the 1950’s almost all US homes had a bomb shelter because engineers had manipulated matter to create devices that could cause massive destruction. But at the same time similar manipulations created methods for treating diseases. Today we are locked down in our homes from an inadvertent virus that threatens our lives. At the same time, biological engineers are manipulating genetic materials to create therapies for curing diseases and new foods.

I am now over 81 years old. In 25 years, I will be over 106 years old, a seemingly unlikely and somewhat unappealing scenario. So, instead of predicting the next 25 years for IBE, I leave you with the following appeal:

- Please do not lose the vision of IBE and deeply reflect possibilities with it.
- Frequently share IBE’s vision widely, and especially with IBE members because most have never heard it.
- Don’t be bashful; reach out to someone who you think will benefit from being active in IBE, invite and engage then in an important project. Publicize widely the wonderful work of its members.
- By doing these you will be inventing a future IBE that would be proudly serving to advance biology-inspired engineering.

My hope is that IBE will provide an environment for biologists and biological engineers to invent a future in which their impact is positive and ubiquitous.

## Footnote

“*As the Institute of Biological Engineering celebrates its 25th anniversary, and Earth encounters daunting biological challenges, the historical and visionary comments of Prof. Verma are timely and remain relevant. We look to the future generation of scientists and engineers to be inspired to invent the next 25 years, to come together to foster cooperation and broad inquiry in biological engineering, and to meet these challenges”* … Dr. Mark Eiteman, IBE President, 2019–2020; Editor-in-Chief, Journal of Biological Engineering, 2009–2016.

*“It is appropriate in the 25th anniversary year of the Institute of Biological Engineering (IBE) to reflect on the origin of IBE, to note its achievements in its first quarter century, and to consider its role in the 25 years to come. At no time has biological engineering been more relevant than it is today, as humanity collectively faces a worldwide pandemic. Engineering biology appears at the forefront of efforts to identify, detect, treat, and ultimately prevent this devastating viral illness. Members of IBE can, and must, participate energetically and purposefully, as individuals and collectively as a society, to expedite overcoming of our present challenge and enable capabilities that minimize risk of infectious disease in years to come. On behalf of IBE, as its president, I invite biological engineers across academia, industry, and government to join us and IBE in this epic challenge”-* Dr. Keith Roper, IBE President 2020–2021.

